# The use of cessation assistance among smokers from China: Findings from the ITC China Survey

**DOI:** 10.1186/1471-2458-11-75

**Published:** 2011-02-02

**Authors:** Jilan Yang, David Hammond, Pete Driezen, Richard J O'Connor, Qiang Li, Hua-Hie Yong, Geoffrey T Fong, Yuan Jiang

**Affiliations:** 1 Department of Health Studies and Gerontology, University of Waterloo, Waterloo, Canada; 2 Department of Health Behavior, Roswell Park Cancer Institute, Buffalo, New York, USA; 3 National Tobacco Control Office, Chinese Center for Disease Control and Prevention, Beijing, China; 4 The Cancer Council Victoria, Melbourne, Australia; 5 Department of Psychology, University of Waterloo, Waterloo, Canada; 6 Ontario Institute for Cancer Research, Toronto, Canada

## Abstract

**Background:**

Stop smoking medications significantly increase the likelihood of smoking cessation. However, there are no population-based studies of stop-smoking medication use in China, the largest tobacco market in the world. This study examined stop-smoking medication use and its association with quitting behavior among a population-based sample of Chinese smokers.

**Methods:**

Face-to-face interviews were conducted with 4,627 smokers from six cities in the ITC China cohort survey. Longitudinal analyses were conducted using Wave 1 (April to August, 2006) and Wave 2 (November 2007 to January 2008).

**Results:**

Approximately 26% of smokers had attempted to quit between Waves 1 and 2, and 6% were abstinent at 18-month follow-up. Only 5.8% of those attempting to quit reported NRT use and NRT was associated with lower odds of abstinence at Wave 2 (OR = 0.11; 95%CI = 0.03-0.46). Visiting a doctor/health professional was associated with greater attempts to quit smoking (OR = 1.60 and 2.78; 95%CI = 1.22-2.10 and 2.21-3.49 respectively) and being abstinent (OR = 1.77 and 1.85; 95%CI = 1.18-2.66 and 1.13-3.04 respectively) at 18-month follow-up relative to the smokers who did not visit doctor/health professional.

**Conclusions:**

The use of formal help for smoking cessation is low in China. There is an urgent need to explore the use and effectiveness of stop-smoking medications in China and in other non-Western markets.

## Background

Cigarette smoking is the single largest preventable cause of premature death worldwide. Up to one half of long-term smokers will die from smoking-related disease [1-3]. Smoking kills more than 5 million people each year and the death toll will double by 2030 if current trends continue [4].

Quitting smoking reduces the risk of smoking-attributable disease. People who quit smoking, regardless of their age, are less likely to die from smoking compared with those who continue to smoke. Smokers who quit before the onset of major smoking-attributable disease avoid most of the excess negative health effects of smoking [5]. For example, smokers who stop smoking before middle age avoid more than 90% of the lung cancer risk caused by smoking [3]. Recent research indicates that quitting smoking even after a diagnosis of lung cancer substantially increases survivorship rates [6]. Therefore, interventions that promote smoking cessation are among the most important public health measures available.

Although more than 80% of the world's smokers live in low and middle income countries, smoking cessation rates in these countries are significantly lower than in high income countries [7]. In high income countries such as the United States, Canada and the Netherlands, former smokers comprise about 30% of the population, in contrast to developing countries such as China, where former smokers account for less than 10% of the population [7].

As the most populous country in the world, China is home to 350 million, or one third of the world's smokers. Although estimates of cessation rates in China vary across studies, they are consistently low. A national study conducted in 1996 concluded that less than 1% of "established" smokers (individuals who had smoked for at least 6 months) had quit for more than two years [8]. A more recent national study conducted in 2003 reported that the "quit rate", defined as smokers who had quit for more than two years among those who had smoked over 100 cigarettes, was 2.5% [9]. Several local studies report higher quit rates -9% in Shanghai [10], 6% in Chengdu [11] and 11% in Jiangsu province [12] -- however, these numbers are consistently lower than rates in Western countries.

Research in Western countries has found that smokers who receive formal cessation assistance are more likely to be successful in quitting [13]. Nicotine Replacement Therapy (NRT), such as nicotine gum, the patch and the nicotine lozenge, increases the odds of quitting compared with placebo between 1.5 to 2 fold in clinical trials [14] and in over-the-counter (OTC) NRT use [15]. Self-help materials, telephone quitlines, and brief advice from health professional, and more intensive behavioural counselling have also been shown to increase odds of quitting [13].

In China, the types and effectiveness of smoking cessation assistance used by smokers remain largely unexplored. Several clinical studies in China have shown that both NRT and professional counselling are effective among adult smokers [16-18], elder smokers who were over 60 years old [19], and young smokers who were under 24 years old [20]. However, the use of cessation assistance in China appears to be low [21]. A recent local study conducted in Huangshi, Hubei province indicated that 98% of smokers who had attempted to quit did so on their own, without formal assistance [22]. Overall, there is little population-based data on stop smoking medication use among Chinese smokers.

The purpose of the current study is to explore health advice from doctors/health professionals, the use of stop smoking methods and smoking abstinence among a population-based sample of Chinese smokers.

## Methods

### Sample

The International Tobacco Control (ITC) China project is a prospective cohort survey designed to evaluate national level tobacco control policies [23]. The ITC China cohort was recruited using a multistage cluster sampling method to obtain a representative sample of adult smokers who were registered residents in the six cities. In each of the six cities, 10 Jie Dao or Street Districts were selected with probability of selection proportional to population size of the Jie Dao. Within each of these Jie Dao, two Ju Wei Hui or residential blocks were selected, again with probability of selection proportional to size. Within each Ju Wei Hui, the addresses of the dwelling units (households) were listed first, and then a sample of 300 addresses were drawn by simple random sampling without replacement. Information on age, gender and smoking status for all adults living in these 300 households is collected. The enumerated 300 households were then randomly ordered, adult smokers were then approached following the randomized order until 40 adult smokers were surveyed. Wave 1(April to August, 2006) of the ITC China survey was conducted in 4,732, and Wave 2(November 2007 to January 2008) was conducted in 4,566. [24]. A total of 3,868 smokers from Wave 1 were successful re-contacted in Wave 2 (83% retention), among which 3,651 were smokers and 217 were abstinent at follow up. Due to missing data, a total 3,824 respondents were included in the analysis, of which 3,616 were smokers and 208 were quitters who reported abstinent at Wave 2.

The ITC China Survey was conducted through face-to-face interviews. All interviewers followed a standard protocol in their interview session with each respondent. Up to four visits to a household were made in order to interview the target person(s) within that household. The enumerators and survey interviewers were trained by the China CDC staff in each city, with support and supervision from the ITC China team.

All training materials and forms for the enumeration process and the survey interviewing were developed together. Several quality control procedures were put in place, including MP3 audio recording smokers' survey by interviewers and checking by quality controller in each city.

All materials and procedures used in the ITC China Survey were reviewed and cleared for ethics by the Research Ethics Board at the University of Waterloo and by the Institutional Review Boards at the China National Centers for Disease Control and Prevention.

### Measures

#### Demographics

Age was categorized as "18-24; 25-39; 40-54; 55+". Education level was categorized into "low" (no education & elementary school); "middle" (Junior high school & high school); and "high" levels (college and higher)". Household monthly income was classified as "low" (3000 yuan and under), "middle" (3001-5000 yuan), and "high" (5001 and above).

#### Smoking status and quitting behaviour

All respondents were smokers (100 cigarettes in lifetime and were smoking at time of Wave 1 survey). Smoking status at Wave 2 was measured by asking "Do you currently smoke or have you quit?" The respondents who self-reported that they had quit were categorized as quitters. Length of smoking abstinence among quitters was measured. Quit attempt was measured by the question of "Since we last talked to you in 2006, how many times have you tried to quit smoking?" Among smokers, the number of quit attempts, date of last quit attempt, and length of abstinence of last quit attempt since the Wave 1 survey were assessed.

#### Smoking cessation assistance

All respondents were asked if they had used stop smoking medications, such as nicotine patch, nicotine gum, Zyban, traditional Chinese medicine and acupuncture since the last survey date.

#### Quit advice from doctors/health professionals

All respondents were asked if they visited a doctor/health professional since the last survey. Respondents who reported visiting a doctor/health professional were asked if they received any advice during their visit. Respondents were categorized into three groups: (1) no visit to doctor/health professional since last survey, (2) visited doctor/health professional but did not receive advice to quit smoking, and (3) visited doctor/health professional and received advice to quit smoking.

### Analysis

All statistical analyses were performed using SAS version 9.2 (SAS Institute Inc., Cary, NC). Analyses were weighted to ensure results were representative of smokers in the six cities included in the ITC China project [23]. Analyses also accounted for the multi-stage sampling design. Unless otherwise noted, all estimates, including percentages, odds ratios (OR) and 95% confidence intervals (95% CI), are weighted estimates while samples sizes are unweighted. Logistic regression was used to test differences in quitting behaviour, use of cessation methods, and visiting a doctor/health professionals and receiving advice to quit during those visits. All odds ratios presented controlled for Wave 1 measures of gender, age, income, education, and daily cigarette consumption.

## Results

### Sample characteristics

As shown in Table 1, more than 95% of respondents were male, consistent with prevalence rates in China. The majority of respondents (83.1%) smoked less 20 cigarettes per day.

**Table 1:** Sample characteristics at Wave 2 of the ITC China Survey (N = 3,824)

	Smokers		"Quitters"		Total	
	%	n	%	n	%	n
**Sex**						
Male	95.2	3442	92.8	193	95.1	3635
Female	4.8	174	7.2	15	4.9	189
						
**Age**						
18-39	16.8	607	10.1	21	16.4	628
40-54	49.8	1801	37.5	78	49.1	1879
55+	33.4	1208	52.4	109	34.4	1317
						
**Income**						
Low	15.6	564	14.9	31	15.6	595
Middle	46.5	1681	48.1	100	46.6	1781
High	31.9	1154	32.7	68	32.0	1222
No answer	6.0	217	4.3	9	5.9	226
						
**Education**						
Low	11.5	416	22.1	46	12.1	462
Middle	67.6	2446	59.1	123	67.2	2569
High	20.9	754	18.8	39	20.7	793
						
**Cigarettes smoked/day**						
0-10	33.2	1200	51.0	106	34.2	1306
11-20	49.9	1804	38.0	79	49.2	1883
21-30	9.2	331	5.3	11	8.9	342
31+	7.7	281	5.8	12	7.7	293

### Quitting behaviour

**Figure 1:**
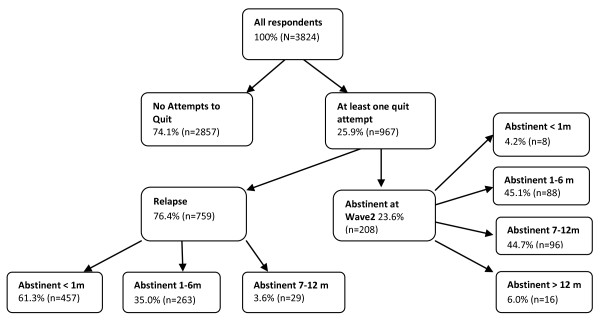
**Quitting behaviour between Wave 1 and 2 (N = 3,824)**. *All estimates presented in Figure 1 are weighted results accounting for multi-stage sampling design.

Figure 1 shows patterns of cessation behaviour between Waves 1 and 2. Among the respondents who self-reported abstinence at Wave 2, less than half of them reported being abstinent for less than 6 months (Figure 1).

#### Quit Attempts

A logistic regression was conducted to examine socio-demographic predictors of quit attempts made between Wave 1 and Wave 2 (Table 2). Smokers age 55 and older had slightly greater odds of making a quit attempt compared to 18-39 year olds (OR = 1.25, 95%CI = 0.95-1.66). High and middle income smokers had significantly lower odds of making quit attempts relative to low income smokers (OR = 0.72 and 0.66, respectively). Compared with smokers who consumed less than 10 cigarettes per day, smokers who had 11-20 cigarette, 21-30 cigarette or more than 31 cigarettes per day had significantly lower odds of making a quit attempt (OR = 0.71, 0.54 and 0.58, respectively).

**Table 2:** Adjusted odds ratios of demographic predictors of quit attempts and abstinence at 18-month follow up.

Covariate	Quit attempts		Abstinence at Wave 2	
		(N = 3,824)		(N = 3,824)
	OR	95% CI	OR	95% CI
**Sex**				
Male	1.00		1.00	
Female	0.96	0.66 - 1.41	1.01	0.49 - 2.08
				
**Age**				
18-39	1.00		1.00	
40-54	0.92	0.72 - 1.17	1.75	0.92 - 3.33
55+	1.25	0.95 - 1.66	**3.16**	**1.76 - 5.68**
				
**Income**				
Low	1.00		1.00	
Middle	**0.66**	**0.51 - 0.86**	1.27	0.69 - 2.34
High	**0.72**	**0.52 - 1.00**	**2.11**	**1.01 - 4.38**
No answer	0.44	0.24 - 0.81	0.70	0.31 - 1.60
				
**Education**				
Low	1.00		1.00	
Middle	1.12	0.84 - 1.49	**0.63**	**0.36 - 1.12**
High	1.10	0.77 - 1.56	**0.45**	**0.25 - 0.83**
				
**Cigarettes smoked/day**				
0-10	1.00		1.00	
11-20	**0.71**	**0.60 - 0.84**	**0.53**	**0.37 - 0.77**
21-30	**0.54**	**0.40 - 0.72**	**0.37**	**0.17 - 0.81**
31+	**0.58**	**0.40 - 0.83**	**0.39**	**0.17 - 0.89**

#### Smoking abstinence

Table 2 also shows the results of a logistic regression predicting abstinence at Wave 2. Smokers who were 55 or older had greater odds of being abstinent at Wave 2 (OR = 3.16) relative to 18-39 year olds. High income smokers had 2.11 times greater odds of being abstinent than low income smokers. Smokers in high education group were less likely to be smoking abstinent compared with low education group (OR = 0.45). Compared with smokers who consumed less than 10 cigarettes per day, those smokers who had 11-20 cigarette; 21-30 cigarette; over 31 cigarette per day had significant lower odds of being abstinent (OR = 0.53, 0.37, 0.39, respectively).

### Visiting doctors/health professionals and quitting advice

A total of 33.6% (n = 1,269) of smokers visited doctors/health professionals in the 18 months since Wave 1. Among those who visited doctors/health professionals, 17.4% of total sample (n = 663) received quitting advice during their visit and 10.9% (n = 418) of those who received advice reported that the advice was helpful.

Logistic regression models estimated the likelihood of visiting a doctor/health professional and receiving advice, attempting to quit, and being abstinent, adjusting for age, gender, income, education and cigarette consumption per day. As Table 3 shows, the odds of visiting a doctor (OR = 1.56, 95%CI = 1.06 - 2.29) and visiting a doctor and receiving advice was greater among smokers aged 55 and older (OR = 2.58, 95%CI = 1.49 - 4.47). High and middle income smokers were also more likely to have visited a doctor (OR = 1.44, 95%CI = 1.05 - 1.97; OR = 1.84, 95%CI = 1.21 - 2.81, respectively), while high income smokers had greater odds than low income smokers of receiving quit advice during a doctor/health professional visit (OR = 1.60; 95%CI = 1.06-2.41).

**Table 3:** Association of visiting the doctors/health professionals and receiving advice to quit smoking, quit attempts, and abstinence among all smokers (n = 3,810)

	Doctor's Visit & Advice to Quit*						Quit attempts			Abstinence at Wave 2		
	Visited doctor, no advice			Visited & advised to quit								
**Covariate**	**OR**	**95% CI**	**P**	**OR**	**95% CI**	**P**	**OR**	**95% CI**	**P**	**OR**	**95% CI**	**P**

**Sex**												
Male	1.00			1.00			1.00			1.00		
Female	1.29	0.81 - 2.06	0.28	0.96	0.64-1.46	0.87	0.97	0.67 - 1.42	0.89	0.99	0.48 - 2.05	0.99
												
**Age**												
18-39	1.00			1.00			1.00			1.00		
40-54	1.02	0.75 - 1.37	0.91	1.31	0.81 - 2.14	0.28	0.91	0.71 - 1.17	0.47	1.71	0.90 - 3.26	0.10
55+	**1.56**	**1.06 - 2.29**	**0.02**	**2.58**	**1.49 - 4.47**	**<0.001**	1.11	0.85 - 1.46	0.446	**2.86**	**1.58 - 5.17**	**<0.001**
												
**Income**												
Low	1.00			1.00			1.00			1.00		
Middle	**1.44**	**1.05 - 1.97**	**0.03**	1.20	0.83 - 1.74	0.33	**0.62**	0.48 - 0.80	**<0.001**	1.23	0.67 - 2.27	0.51
High	**1.84**	**1.21 - 2.81**	**0.01**	**1.60**	**1.06 - 2.41**	**0.03**	**0.65**	**0.47 - 0.91**	**0.01**	1.98	0.95 - 4.12	0.07
No answer	**2.20**	**1.29 - 3.74**	**0.004**	1.59	0.81 - 3.12	0.18	**0.38**	**0.21 - 0.69**	**0.001**	0.65	0.28 - 1.49	0.31
												
**Education**												
Low	1.00			1.00			1.00			1.00		
Middle	0.69	0.46 - 1.04	0.07	0.87	0.63 - 1.20	0.40	1.20	0.89 - 1.64	0.23	0.66	0.37 - 1.16	0.15
High	0.91	0.61 - 1.36	0.64	0.82	0.54 - 1.23	0.34	1.17	0.79 - 1.72	0.44	**0.45**	**0.24 - 0.85**	**0.01**
												
**Cigarettes smoked/day**												
0 - 10	1.00			1.00			1.00			1.00		
11 - 20	**0.68**	**0.52 - 0.89**	**0.01**	**0.74**	**0.55 - 0.99**	**0.04**	**0.74**	**0.62 - 0.89**	**0.001**	**0.56**	**0.38 - 0.81**	**0.002**
21 - 30	0.66	0.43 - 1.01	0.06	0.99	0.68 - 1.46	0.97	**0.52**	**0.39 - 0.70**	**<0.001**	**0.37**	**0.17 - 0.81**	**0.01**
31+	**0.55**	**0.32 - 0.95**	**0.03**	**0.67**	**0.47 - 0.96**	**0.03**	**0.58**	**0.41 - 0.81**	**0.001**	**0.42**	**0.19 - 0.96**	**0.04**
												
**Doctor's advice to quit**												
Did not visit doctor	N/A						1.00			1.00		
Visited doctor, no advice							**1.60**	**1.22 - 2.10**	**<0.001**	**1.77**	**1.18 - 2.66**	**0.01**
Visited & advised to quit							**2.78**	**2.21 - 3.49**	**<0.001**	**1.85**	**1.13 - 3.04**	**0.02**

* Based on a multinomial logit model using doctor/health professional visit and advice to quit as the outcome variable, where "Did not visit doctors/health professionals since last survey" was used as the reference category.

Smokers who had visited a doctor/health professional and received advice since baseline had significant greater odds of making a quit attempt (OR = 2.78; 95%CI = 2.21-3.49) and being abstinent (OR = 1.85, 95%CI = 1.13-3.04) at Wave 2, compared to smokers who did not visit a doctors/health professional since Wave 1. Even smokers who visited a doctor/health professional but did not receive advice, had significantly greater odds of making a quit attempt (OR = 1.60; 95%CI = 1.22-2.10) and being abstinence (OR = 1.77, 95%CI = 1.18-2.66), relative to the smokers who did not visit a doctor/health professional. Among smokers who visited a doctor/health professional, those who had received quitting advice during the visit were significantly more likely to have made a quitting attempt (OR = 1.74, 95%CI = 1.31-2.30), but not significantly more likely to be abstinent at follow-up (OR = 1.04, 95%CI = 0.59-1.86; data not shown in table).

### Use of stop smoking medications

Of the smokers who attempted to quit between Waves 1 and 2 (n = 967), 5.8% (n = 62) reported using NRT and/or Zyban. A total of 2.1% (n = 25) reported using "traditional Chinese medicine" and less than one percent (n = 10) reported using acupuncture as a smoking cessation aid. Among all the smokers who attempted to quit between two waves, approximately 1.4% (n = 17) reported using more than one stop smoking medications.

#### Predictors of stop-smoking medication use

Logistic regression models were conducted to examine demographic predictors of NRT and/or Zyban use, and "traditional Chinese medicine" and/or acupuncture use. No significant association was observed for NRT/Zyban use across age, gender, income, education, and cigarettes per day, and the number of prior quit attempts. However, smokers with higher education were 4.28 times more likely to use traditional Chinese medicine/acupuncture (95%CI = 1.41-12.97) than smokers in middle education level.

#### Stop-smoking medications and abstinence

Among those who attempted to quit and exclusively used NRT and/or Zyban as stop smoking assistance (n = 50), only 3.3% (n = 2) reported being abstinent at Wave 2. In contrast, among smokers who tried to quit smoking on their own (n = 885), 24.8% (n = 200) were smoking abstinent at Wave 2. As Table 4 shows, smokers who used NRT were significantly less likely to be abstinent at Wave 2 compared with those attempting to quit without assistance after adjusting for age, gender, education, income, and cigarette consumption per day and prior quit attempts (OR = 0.11, 95%CI = 0.03-0.46). Among those who quit using traditional Chinese medicine (n = 13) exclusively, 16.4% (n = 2) reported abstinence at follow-up. There was no significant difference in abstinence between quitters who reported using traditional Chinese medicine and acupuncture, and those who reported no cessation assistance (see Table 4).

**Table 4:** Association between stop-smoking medications and abstinence among respondents that made at least one quit attempt (n = 955)

Covariates	OR	95% CI	P level
**Sex**			
Male	1.00	-	
Female	1.39	0.54 - 3.56	0.50
			
**Age**			
18-39	1.00	-	
40-54	**2.06**	**1.14 - 3.72**	**0.02**
55+	**3.07**	**1.63 - 5.78**	**<0.001**
			
**Income**			
Low	1.00	**-**	
Middle	1.97	0.95 - 4.11	0.07
High	**3.62**	**1.68 - 7.78**	**0.001**
No answer	1.18	0.40 - 3.49	0.77
			
**Education**			
Low	1.00	-	
Middle	**0.50**	**0.26 - 0.97**	**0.04**
High	**0.29**	**0.14 - 0.62**	**0.001**
			
**Cigarettes smoked/day**			
0-10	1.00	-	
11-20	0.64	0.40 - 1.02	0.06
21-30	0.51	0.19 - 1.42	0.20
31+	0.63	0.26 - 1.55	0.32
			
**Prior Attempts**			
None	1.00	-	
Once	0.99	0.61 - 1.62	0.98
2-5 times	0.87	0.25 - 3.12	0.84
6-10 times	1.17	0.42 - 3.23	0.76
			
**Cessation assistance**			
No assistance	1.00	-	
NRT or Zyban	**0.11**	**0.03 - 0.46**	**0.002**
Used traditional Chinese medicine or acupuncture	0.61	0.15 - 2.43	0.48

## Discussion

Given the looming health burden from 350 million tobacco users in China, efforts to increase smoking cessation rates are among the most important public health measures in China. The current study indicates that the prevalence of smokers actively trying to quit smoking in China is significantly lower than in Western countries. Approximately one quarter of Chinese smokers reported trying to quit in the last 18 months compared to almost half of smokers in the US and Canada [13,25]. These findings are consistent with reports of lower motivation to quit among Chinese smokers [26], which may reflect lower levels of health knowledge or more supportive social norms towards smoking in China [27].

According to available research, about half of quitters who are abstinent for less than 6 month will subsequently relapse, and one-fifth of those abstinent for six to twelve month will relapse [28], Therefore, the proportion of smokers in the current study who will achieve long term abstinence is likely to be well below the 6% of smokers who reported abstinence at follow-up. Current estimates indicate that annual quit rate in low and middle income countries is typically 4% to 7% [13,15]. Therefore, the actual quit rate in China is likely lower than that in high income countries.

Low income smokers in the current study were more likely to make quit attempts than smokers with higher income; however, lower income smokers were less likely to report smoking abstinence. In other words, lower income smokers may be have greater interest in quitting but have lower capacity to maintain abstinence. This finding suggests that there is a need in China for population-based smoking cessation interventions to ensure that smokers from low socioeconomic groups have greater access to effective forms of cessation assistance.

Visiting doctors/health professionals was associated with greater attempts to quit smoking and abstinence. However, receiving advice on quitting during the doctor/health professional visit was not associated with higher levels of abstinence among those attempting to quit. Several factors may account for this finding. First, research suggests that smoking prevalence is as high as 23% among Chinese physicians and 41% among male physicians. Knowledge of the health effects of smoking also appears to be low among physicians: in 2007, only two thirds of Chinese physicians reported that smoking causes heart disease [29]. This lack of knowledge may limit the capacity of health professionals to effectively advise smokers. Second, approximately two thirds of Chinese physicians do not believe that smokers would follow their cessation advice [29]. Despite this, a majority of smokers who visited a doctor/health professional in the current study received cessation advice. This finding highlights the need to examine the content of health professionals' advice to a greater extent.

Use of stop smoking medications among Chinese smokers was extremely low--less than 6% for NRT and Zyban combined. Given that the current sample is drawn from large urban centers in China, NRT use is likely to be even lower among smokers in rural areas and entire population in China. The current findings are consistent with recent evidence indicating that few smokers regard NRT as an effective and viable smoking cessation aid given the high price and relatively low levels of marketing in China [30].

Evidence from developed countries supports the effectiveness of stop-smoking medications such as NRT and Zyban, which are among the "first-line" treatments for smoking cessation in Western countries [13,14]. However, the results from the current study suggest that Chinese smokers who quit using NRT were significantly less likely to be abstinent at follow-up, compared with those who quit without stop-smoking medications. Although several clinical trials support the effectiveness of NRT among Chinese smokers [16-18], our findings are the first "real-world" evaluation of NRT among Chinese smokers to our knowledge. These preliminary findings suggest no positive impact from NRT among Chinese smokers after previous quit attempts were controlled after adjusting for important factors such as prior quit attempts [31]. Nevertheless, the findings highlight the need for additional population-based study to examine the effectiveness of stop-smoking medications outside of clinical trials in low and middle income countries. Even in high income Western countries, some within the tobacco control community have questioned the population-level benefit of stop-smoking medications [32]. The question of whether low and middle income countries such as China should invest heavily in smoking cessation services and aggressively promote greater use of pharmacotherapy is also being addressed as part of the World Health Organization's Framework Convention on Tobacco Control (FCTC), the world's first public health treaty. Article 14 of the FCTC treaty requires countries to promote the use of smoking cessation services; however, the extent to which low and middle income countries should following the model of countries such as the United Kingdom and South Korea and invest in cessation clinics and subsidies for medications is a pressing issue for which little evidence exists to guide regulations.

## Limitations

This study has several limitations common to survey research, including the limitations of self-reported data and potential sample bias. In particular, abstinence was self-reported at follow-up and not biochemically verified in any way. A measure of continuous abstinence, such as sustained 6-month abstinence, would yield lower estimates of cessation activity. However, the response rates of the ITC China are significantly higher than those commonly reported in Western countries and the cohort design of this study is a considerable strength.

## Conclusions

Tobacco control activities in China have lagged behind most Western countries. China has recently taken several important recent steps, including ratifying the WHO FCTC, implementing smoke-free policies, as well as launching several media campaigns. However, the current study indicates that rates of smoking cessation in China are considerably lower than in Western countries. Very few Chinese smokers use formal assistance when trying to quit, including stop-smoking medications. In addition, preliminary findings suggest that smokers who used NRT or Zyban were less likely to quit than Chinese smokers who reported quitting without assistance. The findings highlight the urgent need to better understand patterns of quitting and the use of cessation assistance in China, as well as other low and middle income countries which bear the overwhelming global burden of disease from tobacco use.

## Compteting interests

The authors declare that they have no competing interests.

## Authors' contributions

JLY and DH have made major contributions to research conception and design and were responsible for preparing the manuscript draft. PD conducted the statistical analyses and participated in interpreting data analyses. All authors have read and provided comments during the manuscript preparation and approved the final manuscript.

## Pre-publication history

The pre-publication history for this paper can be accessed here:

http://www.biomedcentral.com/1471-2458/11/75/prepub

## References

[B1] Federal Register U.S. Department of Health and Human Services, Food and Drug Administration Regulations restricting the sale and distribution of cigarettes and smokeless tobacco to protect children and adolescents 1996 44395 445 61 Fed Reg 20383919

[B2] Thun MJ Day-Lally C Myers DG Trends in tobacco smoking and mortality from cigarette use in Cancer Prevention Studies I (1959 through 1965) and II (1982 through 1988) 1997 National Cancer Institute. Smoking and Tobacco Control Monograph No. 8. U.S. Department of Health and Human Services, Public Health Service, National Institutes of Health, National Cancer Institute 305 82 NIH Publication No. 97-4213

[B3] Peto R Darby S Deo H Silcocks P Whitley E Doll R Smoking, smoking cessation, and lung cancer in the UK since 1950: combination of national statistics with two case-control studies Br Med J 2000 321 323 329 10.1136/bmj.321.7257.323 PMC27446 10926586

[B4] WHO report on the Global Tobacco Epidemic 2008, the MPOWER package 2008 Geneva, World Health Organization http://whqlibdoc.who.int/publications/2008/9789241596282_eng.pdf

[B5] Doll R Peto R Boreham J Sutherland I Mortality in relation to smoking: 50 years' observations on male British doctors Br Med J 2004 328 1519 1527 10.1136/bmj.38142.554479.AE PMC437139 15213107

[B6] Parsons A Daley A Begh R Aveyard P Influence of smoking cessation after diagnosis of early stage lung cancer on prognosis: systematic review of observational studies with meta-analysis BMJ 2010 340 b5569 10.1136/bmj.b5569 20093278 PMC2809841

[B7] Storr CL Cheng H Alonso J Angermeyer M Bruffaerts R Smoking estimates from around the world: Data from the first 17 participating countries in the World Mental Health Survey Consortium Tob Con 2009 10.1136/tc.2009.032474 PMC4124902 19965796

[B8] Yang GH Fan LX Tan J Smoking in China: Findings of the 1996 National Prevalence Survey JAMA 1999 282 1247 1253 10.1001/jama.282.13.1247 10517427

[B9] Qian JC Rao KQ Gao J Reasons of quitting and relapsing smoking and analysis of quitter's health status Chin J Health Stat 2009 26 150 153

[B10] Xu JY Li XJ Yao HH Smoking and Passive Smoking among Residents in Shanghai Chin J Prev Contr Chron Dis 2009 17 3234 3236

[B11] Jia Y Du CH Wei YL Wang Q Zhang WJ Liao J Quitting behavior among smokers in Chengdu from 1996-2002 Chin J Prev Contr Chron Dis 2005 13 237 238

[B12] Pan XQ Lui SR Xiang QY Cheng JY Wu M Qin Y Behaviour of smoking cessation and received service among male smokers in Jiangsu Province Chin J Health Educ 2009 125 424 426

[B13] Fiore MC Jae'n CR Baker TB Treating Tobacco Use and Dependence: 2008 Update Clinical Practice Guideline 2008 U.S. Department of Health and Human Services: Rockville MD

[B14] Silagy C Lancaster T Stead L Mant D Fowler G Nicotine replacement therapy for smoking cessation Cochrane Database of Systematic Reviews 2004 3 CD000146 10.1002/14651858.CD000146.pub2 15266423

[B15] Hughes JR Shiffman S Callas P Zhang J A meta-analysis of the efficacy of over-the-counter nicotine replacement Tob Con 2003 12 21 27 10.1136/tc.12.1.21 PMC1759113 12612357

[B16] Lam T-H Abdullah AS Chan SS Hedley AJ Adherence to nicotine replacement therapy versus quitting smoking among Chinese smokers: a preliminary investigation Psychopharmacology 2005 177 400 408 10.1007/s00213-004-1971-y 15289997

[B17] Yu HX Zang YN Lin JT The effect of the abstinence from smoking with nicotine replacement therapy combining with psychological and ehavior intervention Chin J of Reh Med 2006 21 1104 1106

[B18] Sun H-Q Guo S Chen D-F Jiang Z-N Liu Y Di X-D Family Support and Employment as Predictors of Smoking Cessation Success: A Randomized, Double-Blind, Placebo-Controlled Trial of Nicotine Sublingual Tablets in Chinese Smokers Am J Drug Alcohol Abuse 2009 35 183 188 10.1080/00952990902839794 19462302

[B19] Abdullah A-S Lam T-H Chan S Leung G-M Chi I Ho W Effectiveness of a mobile smoking cessation service in reaching elderly smokers and predictors of quitting BMC Geriatr 2008 8 25 10.1186/1471-2318-8-25 18837985 PMC2570661

[B20] Abdullah A-S Lam T-H Chan S Hedley A-J Smoking cessation among Chinese young smokers: does gender and age difference matters and what are the predictors? Addict Behav 2006 31 913 21 10.1016/j.addbeh.2005.08.009 16150551

[B21] Zhang YD Xian MJ Li GQ Smoking cessation among smokers in Conghua. *Henan* J Prev Med 2003 14 41 43

[B22] Luo CL Smoking and quitting among smokers in different occupations in Huangshi? J of Pub Health and PrevMed 2009 20 126

[B23] Wu CB Thompson ME Fong GE Jiang Y Yang Y Feng GZ Li Q Methods of the International Tobacco Control (ITC) China survey Tob Con 2010 in press 10.1136/tc.2009.029900 PMC2975999 19648134

[B24] Wave 2 (2007-2008) ITC China Technical Report http://www.itcproject.org/library/countries/itcchina/reports/cn2techrptrevjul72010pdf

[B25] Leatherdale S Shields M Smoking cessation: intentions, attempts and techniques Statistics Canada, Catalogue no. 82-003-XPE • Health Reports 2009 20 19813437

[B26] ITC Project ITC China Survey Summary 2009 University of Waterloo, Waterloo, Ontario, Canada, and Chinese Center for Disease Control and Prevention, Beijing, China

[B27] Yang JL Hammond D Driezen P Fong G Jiang Y Health knowledge and perception of risk among Chinese Smokers and Non-Smokers: Findings from the ITC China Survey Tob Con 2010 in press 10.1136/tc.2009.029710 PMC5654747 20935195

[B28] Herd N Borland R Hyland A Predictors of smoking relapse by during of abstinence: finding from the International Tobacco Control (ITC) Four Country Survey Addict 2009 104 2088 2099 10.1111/j.1360-0443.2009.02732.x PMC4517970 19922574

[B29] Jiang Y Michael K Ong Elisa K Tong Chinese physicians and their smoking knowledge, attitudes, and practices Am J Pre Med 2007 33 15 22 10.1016/j.amepre.2007.02.037 PMC2800817 17572306

[B30] Zhong ZH Thoughts from three international medical companies coming into Chinese smoking cessation medication marketing within next two years J Chi Pres Drug 2009 89 15

[B31] Balmford J Borland R Burney S The role of prior quitting experience in the prediction of smoking cessation Psychol Health 2009 14 1 14 10.1080/08870440902866878 20204954

[B32] MacKenzie R Chapman S The Global Research Neglect of Unassisted Smoking Cessation: Causes and Consequences PloS Med 2010 7 e1000216 10.1371/journal.pmed.1000216 20161722 PMC2817714

